# Celebrating the Fabric of Commonplace Society

**DOI:** 10.3201/eid2111.AC2111

**Published:** 2015-11

**Authors:** Byron Breedlove, Nkuchia M. M’ikanatha

**Affiliations:** Centers for Disease Control and Prevention, Atlanta, Georgia, USA (B. Breedlove); Pennsylvania Department of Health, Harrisburg, Pennsylvania, USA (N.M. M’ikanatha)

**Keywords:** art science connection, emerging infectious diseases, celebrating the fabric of commonplace society, Ebola, batik, art and medicine, Mozambique, about the cover

**Figure:**
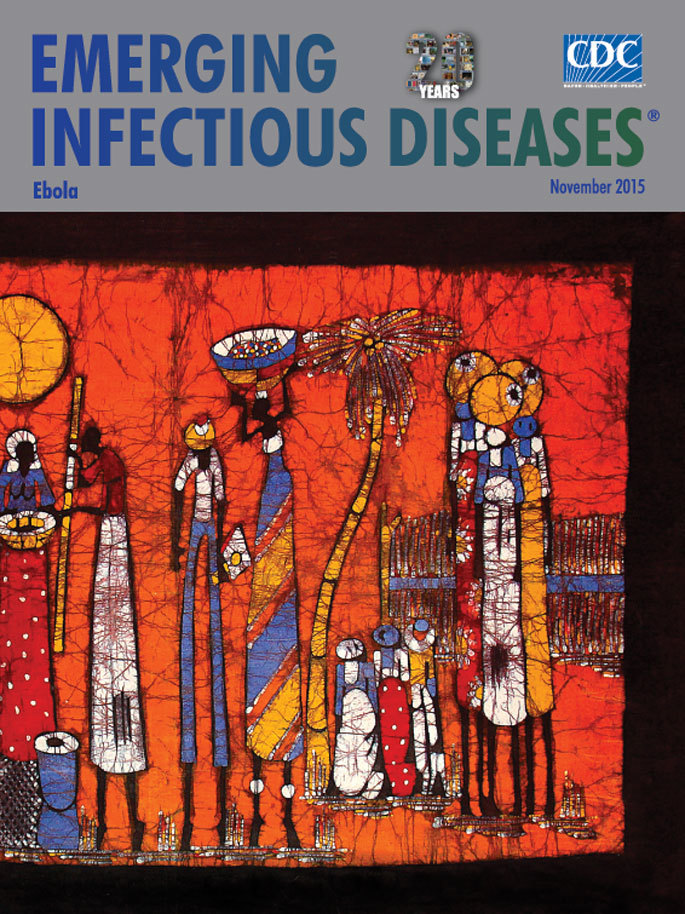
**Unknown (contemporary). Cotton canvas, pastel, acrylic, ink. 39 × 59 in/99.06 × 149.86 cm.** Parque dos Continuadores, Maputo, Mozambique. Personal Collection, Philip Lederer, USA. Photograph by David Swerdlow.

Batik is an ancient creative art that uses wax and dye to decorate cloth. Although evidence of this art has been documented in many parts of the world, probably its origin is in the Island of Java, Indonesia. The word batik is derived from the Indonesian word *ambatik*, which means a cloth with little dots. Batik involves brushing or drawing molten wax onto designated areas of a cloth so that those areas can then resist coloration. Next, the cloth is dyed and the waxed sections retain their original color. After drying the cloth, typically by sunlight, the artist repeats this process by waxing, dyeing, and drying to create more intricate and colorful designs. In the last step, the artist applies the final dye and removes the wax, yielding a beautifully decorated cloth (batik) that may be displayed, used, or worn.

Although contemporary batik draws from this traditional process, it has changed in several ways. Today, batik artists use other forms of dyeing, different tools, and new recipes for the wax; they also use an expanded range of materials, including silk, cotton, wool, leather, paper, wood, and ceramics. Moreover, cultural influences on batik patterns and motifs are noticeable in many parts of world, including communities in Asia and Africa. In 2009, UNESCO designated handcrafted batik an intangible cultural value passed down for generations. That designation symbolizes not only our shared creativity but also our diversity—representing what connects humanity to the past, present, and future. The untitled contemporary batik displayed on this month’s cover came from a street market in Maputo, Mozambique, in April 2012. In Maputo and other cities in Africa, including Freetown in Sierra Leone, batiks are sold on street corners; often the artist, including the creator of this month’s cover, is unknown.

Mozambican batiks frequently express daily scenes with striking colors—that in this example draw you to elongated figures, dazzling coloration, flattened perspective, and a celebratory occasion, in time of tranquility. Festooned in an array of bright color, the villagers conveying various foods converge toward a pair of figures, one hovering over a large blue mortar, the other holding a long pole. The shorter figures to the right may be kneeling in reverence; the standing figures all appear to be on their tiptoes—perhaps it has recently rained. There is no infusion of allegory, certainly no irony, though there may be intended subtleties that convey cultural messages in the choice of colors and patterns.

Because of their universal recognition, batiks can represent commonplace life, culture, and society, yet they also highlight the many bright moments that stand out from the daily rigors, chores, and routines. These striking clothes literally embody the fabric of everyday life, which at times succumbs to natural and anthropogenic forces. Thus, batiks serve as metaphors for what is lost in a population overpowered by natural or other emerging threats—this undoubtedly happened during the Ebola epidemic in West Africa. Joanne Liu, International President of Médecins Sans Frontières, stated that when she first arrived in West Africa in 2014, Ebola “was destroying families and ripping apart the very fabric of society, while national authorities and a handful of aid organisations desperately struggled against this unrelenting, invisible foe.”

The World Health Organization has documented more than 28,000 reported cases and more than 11,000 reported deaths attributed to Ebola. According to the United Nations Development Group, “West Africa as a whole may lose an average of at least US$3.6 billion per year between 2014 and 2017, due to a decrease in trade, closing of borders, flight cancellations and reduced Foreign Direct Investment and tourism activity, fueled by stigma.” The close connection among African nations means that the Ebola crisis also affects those countries in which disease incidence was low or zero. Countries beyond Africa monitored international travelers in addition to other measures implemented in response to the Ebola epidemic, demonstrating that infectious diseases are not contained by borders or geography.

Through a concerted effort, the fabric of society in West Africa is in the process of being repaired, bringing back stability and normalcy. The possibility of a vaccine for Ebola tantalizes the world while attention to global health security has been reignited. The colorful batik on this month’s cover reminds us to keep celebrating the quotidian.
